# Diabetic Osteoporosis: A Review of Its Traditional Chinese Medicinal Use and Clinical and Preclinical Research

**DOI:** 10.1155/2016/3218313

**Published:** 2016-09-06

**Authors:** Rufeng Ma, Ruyuan Zhu, Lili Wang, Yubo Guo, Chenyue Liu, Haixia Liu, Fengwei Liu, Hongjun Li, Yu Li, Min Fu, Dongwei Zhang

**Affiliations:** ^1^Preclinical Medicine School, Beijing University of Chinese Medicine, Beijing 100029, China; ^2^Chinese Materia Medica School, Beijing University of Chinese Medicine, Beijing 100029, China; ^3^Henan Luoyang Orthopedic Traumatological Hospital, Luoyang 471000, China; ^4^The Research Institute of McGill University Health Center, Montreal, QC, Canada H4A 3J1; ^5^Diabetes Research Center, Beijing University of Chinese Medicine, Beijing 100029, China

## Abstract

*Aim*. The incidence of diabetic osteoporosis (DOP) is increasing due to lack of effective management over the past few decades. This review aims to summarize traditional Chinese medicine (TCM) suitability in the pathogenesis and clinical and preclinical management of DOP.* Methods*. Literature sources used were from Medline (Pubmed), CNKI (China Knowledge Resource Integrated Database), and CSTJ (China Science and Technology Journal Database) online databases. For the consultation, keywords such as diabetic osteoporosis (DOP), TCM, clinical study, animal experiment, toxicity, and research progress were used in various combinations. Around 100 research papers and reviews were visited.* Results*. Liver-spleen-kidney insufficiency may result in development of DOP. 18 clinical trials are identified to use TCM compound prescriptions for management of patients with DOP. TCM herbs and their active ingredients are effective in preventing the development of DOP in streptozotocin (STZ) and alloxan as well as STZ combined with ovariectomy insulted rats. Among them, most frequently used TCM herbs in clinical trials are Radix* Astragali*, Radix et Rhizoma* Salviae Miltiorrhizae*, Radix* Rehmanniae Preparata*, and Herba* Epimedii*. Some of TCM herbs also exhibit toxicities in clinical and preclinical research.* Conclusions*. TCM herbs may act as the novel sources of anti-DOP drugs by improving bone and glucolipid metabolisms. However, the pathogenesis of DOP and the material base of TCM herbs still merit further study.

## 1. Introduction

Diabetic osteoporosis (DOP) is a chronic bone metabolic disease induced by diabetes mellitus (DM), and its pathogenesis involves an increase in osteoclast activity, a decrease in osteoblast activity, aggravation of bone microcirculation, an increase in adipogenic differentiation of mesenchymal multipotential stem cells (MMSCs), and an increase in advanced glycation end products (AGEs) [1, 2]. With the increasing incidence of DM in the world, the number of patients with DOP increased accordingly [3]. Clinical studies have shown that about 1/2 to 2/3 of diabetic patients suffered from decreasing bone strength and/or increasing incidence of fractures, of whom nearly 1/3 were diagnosed as osteoporosis [4]. In recent years, traditional Chinese medicine (TCM) has attracted increasing attention in prevention and treatment of DOP. This review highlights the pathogenetical, clinical, and experimental advances in management of DOP in TCM and also provides a strong scientific evidence for understanding and improving the treatment of DOP.

## 2. DOP Pathogenesis in TCM

DOP belongs to the secondary osteoporosis, whose concept was first proposed in 1948 [2, 46]. In TCM, there is no corresponding term to describe DOP. However, the clinical performances are similar to the TCM symptoms of “atrophic debility of bones,” “*Gubi,*” “dryness of bone,” or “pain in waist and lower extremities” [47]. According to TCM theories and clinical symptoms of DOP, the etiology of DOP is mainly attributed to the insufficiency of the spleen, liver, and kidney. In traditional Chinese physiology, the spleen (as is well known, all the organs in TCM bear the least resemblance to its western counterpart) is assumed to be responsible for assimilation of nutrients and maintenance of physical strength. The spleen governs blood, digestion, transformation, and transportation in the body [48]. So the spleen has the ability of transforming food into* Qi* and blood and transporting them throughout the body. Proper functioning of the organ is essential to maintain muscle mass and strengthen limbs. Also, in traditional Chinese physiology, the liver (*Gan*) stores blood, ensures the smooth flow of* Qi*, and controls the action and nutrition of tendon [49]. And the kidney (*Shen*) stores essence and controls the growth and development of bone [50, 51]. Meanwhile,* Qi* (*Qi* is the term used in TCM to describe the body's vital life energy [52]) deficiency and blood stasis also contributed to the development of DOP.

*Xiao*-*Ke* is an official terminology for diabetes in TCM [53]. TCM believes that* Xiao*-*Ke* mainly resulted from the dysfunctions of kidney, spleen, and liver [54]. As kidney controls bone growth and development via neuroendocrine-immune network and osteoclast regulatory pathway osteoprotegerin- (OPG-) receptor activator of nuclear factor kappa-B ligand- (RANKL-) RANK [51, 55], the deficiency of the kidney caused by* Xiao-Ke* further results in bone malnutrition. The liver dredges and smooths the route of something and produces blood by promoting circulation of blood and metabolism of body fluid and assisting the spleen and the stomach to digest food (http://old.tcmwiki.com/wiki/the-liver). The loss of liver* Yin* in the development of* Xiao-Ke* causes blood deficiency, which leads to blood stasis, tendon, and vessels undernutrition [56]. The spleen has the function to transport and transform nutrients by digesting food, absorbing nutrients of food and water, and then transporting them to the heart and the lung (http://old.tcmwiki.com/wiki/the-spleen). If the essence transportation and transformation fails in spleen, dampness and circulation disorders of* Qi* and blood will occur to the body [57]. Further, the bone is loss of nutrition which may cause insufficiency of bone marrow and bone pain [58]. The codysfunctions of liver, spleen, and kidney lead to* Qi* deficiency and blood stasis, which further contributes to insufficiency of blood and essence [57, 59]. All the abnormalities lead to bone lesions and occurrence of DOP. The views on the pathogenesis of DOP have been also discussed in the ancient Chinese medicine works, such as “Inner Canon of* Huangdi,*” “Clinic Guideline of Medical Records,” and “*Xue Zheng Lun.*” Therefore, prevention and treatment of DOP should focus on regulation and tonification of the liver, spleen, and kidney. The proper function of liver, spleen, and kidney promotes* Qi* to disperse stagnation and reinforce kidney to replenish* Yin*, which beneficially contributes to improving bone metabolism.

In recent years, great achievements have been made in prevention and treatment of DOP in TCM. The results have demonstrated that TCM herbs not only had the abilities of increasing the bone quality by enhancing the mechanical strength of bone but also exhibited the effect on improving the primary disease by regulating glycaemia metabolism. Next, we review the clinical and preclinical research advances of using TCM in the treatment of DOP.

## 3. Clinical Advances of DOP in TCM

As mentioned previously, the clinical guidelines for TCM treatment of DOP are tonifying liver, spleen, and kidney as well as promoting* Qi* flow and removing blood stasis. We summarized the TCM compound prescriptions used in the clinical trials of DOP as shown in Table 1. Till now, there are around 18 TCM prescriptions that have been used in managing patients with DOP, in which clinical studies have been performed by different investigators with various prescriptions. Zhang et al. [5] found that Tanggukang treatment reduced serum malondialdehyde (MDA) and blood glucose and increased the activity of superoxide dismutase (SOD) that contributed to controlling the glycaemia and suppressing osteoporosis in patients with DOP. In addition, both Tanggukang [6] and Qianggubao granule treatments also had significant effects on patients with DOP evidenced by improving calcium and phosphorus metabolism and hydroxyproline level as well. In addition, Ji et al. [7] found that Bushen Huoxue decoction treatment not only controlled glycaemia and lipidemia but also obviously improved bone mineral density (BMD) in patients with DOP. Shang et al. [8, 9] found that Bushen Tongluo decoction had the ability of improving BMD and reducing the risk of bone fractures in patients with DOP. Gong and Li [10] found that Jiawei Qing'e pill treatment improved DOP patients in blood glycosylated hemoglobin (HbA1C), calcium and phosphorus level, and fasting urine Ca/creatine ratio which beneficially contributed to the balance of bone metabolism. Yushanjiang and Halida [11] showed that the Xihongkang treatment prevented the development of DOP by tonifying* Qi*, nourishing* Yin*, and promoting blood circulation. Deng [12] demonstrated that Yishen Zhuanggu decoction treatment increased serum bone gla-protein (BGP) and 1,25-(OH)_2_D_3_ levels and BMD value in patients with DOP.

Furthermore, treatments with TCM formulas, such as Huqian pills [13], Gusong decoction [14, 15], Tangmaikang granules [16], Bushen Huoxue decoction [17], Jiawei Gutongxian capsule [18], Qianggubao granule [19], Jiangu decoction [20], Jiangtang Bushen formulation [21], Huolisu oral liquid [22], Migu decoction [23], and Xianling Gubao [24], have also been demonstrated to improve BMD, decrease serum C-reactive protein (CRP) level, and attenuate lumbar pain evidenced by decreasing visual analogue scale pain score in vertebra and back in the patients with DOP.

Most of prescriptions used in management of DOP consist of 4 to 14 herbs. Eight of most appearing Chinese herbs in the clinical studies are compiled in Table 2. All of these herbs possess the functions of invigorating* Qi*, activating blood circulation, and replenishing vital essence to tonify kidney [60]. The applications also conform to the TCM pathogenesis of DOP. The prescriptions are helpful for the investigators to screen the active ingredients against DOP from these herbs in the future.

Based on the listed prescriptions from Table 1, it is found that TCM treatments improved DOP through multichannel and different levels in the clinical trials. And the most commonly used herbs are Radix* Astragali*, Radix et Rhizoma* Salviae Miltiorrhizae*, Herba* Epimedii*, and Radix* Rehmanniae* Preparata. These four herbs have also been well evidenced to play a beneficial role in the treatment of diabetes by inhibiting AGEs formation [61], improving mitochondrial function and biogenesis, interfering with cell cycle [62], increasing insulin sensitivity and glycogen synthesis [63–65], and improving 5′ adenosine monophosphate-activated protein kinase signaling [66, 67] as well as regulating tissue regeneration and angiogenesis to promote diabetic foot ulcer healing [68]. Meanwhile, these four herbs were also demonstrated to exhibit antiosteoporotic effect by inhibiting osteoclastogenesis and promoting osteoblastogenesis through regulating mitogen-activated protein kinase [69], Wnt/*β*-catenin, bone morphogenetic proteins/SMAD, and OPG/RANKL/cathepsin K/nuclear factor kappa-B (NF-*κ*B) signaling [26, 70, 71].

However, the current clinical studies are limited to small scale of samples and regional hospitals. Meanwhile, the clinical trials do not fully conform to the rule of randomized, double-blind, and parallel group which markedly impacted the correct evaluation of clinical research results. In addition, clinical observation indexes are limited more in BMD than in bone strength and fracture incidence. The stability and repeatability of the compound preparations used in clinical trials also remain open and should be studied further. Therefore, the credibility of the results needs to be further improved. So it is not conducive to developing standardized diagnosis method and treatment program as well as the development of new drugs.

## 4. Preclinical Advances of DOP in TCM

### 4.1. The Applications of Single Chinese Herbs in the Treatment of DOP in Animal Experiments

Single herbs or their extracts have been extensively studied in the DOP animal models (Table 3). In an experiment performed by Zhang et al., the water fraction of Fructus* Ligustri Lucidi* (FLL) ethanol extract (WF-EE, 574 mg/kg, i.g.) has been demonstrated to improve the trabecular bone deteriorations and inhibit hypercalciuria and increase serum parathyroid hormone (PTH) and fibroblast growth factor-23 in streptozotocin (STZ, 40 mg/kg) induced DBA/2J mice [25]. The underlying mechanism of WF-EE treatment may be attributed to increase of gene expressions of transient receptor potential vanilloid (TRPV) and calcium binding protein 9 K (CaBP-9K) in duodenum in DOP mice. Further, using alloxan (200 mg/kg) induced diabetic rats, Radix et Rhizoma* Salviae Miltiorrhizae* treatment (5 g/kg for 8 weeks) [26, 27] significantly improved serum calcium, alkaline phosphatase (ALP), BGP, and tartrate-resistant acid phosphatase levels and increased BMD value in alveolar bone osteoporosis. In addition, treatment with tetramethylpyrazine (100 mg/kg for 15 weeks, the active ingredients isolated from Rhizoma* Chuanxiong*) [28] markedly increased bone hydroxyproline and collagen levels in streptozotocin (STZ, 60 mg/kg) induced diabetic rats. Treatment with the alcohol extracts of* Eucommia ulmoides* Oliv. leaves [29] (6 g/kg) improved serum estradiol and increased BMD values in STZ (50 mg/kg) induced diabetic and ovariectomized (OVX) rats. Fructus* Ligustri Lucidi* [72], Rhizoma* Chuanxiong* [73], the leaves of* Eucommia ulmoides* Oliv. [74], and Radix et Rhizoma* Salviae Miltiorrhizae* [75] have been demonstrated with the function of tonifying kidney and promoting blood circulation.

Treatment with puerarin, the active substance isolated from Radix* Puerariae* with the function of improving blood circulation [76], significantly increased ALP level (40 *μ*M for 48 hours, i.p.) [77], decreased expression of caspase-3 in osteoblasts (80 mg/kg/day for 6 weeks; i.p.) [30], and reduced blood glucose and improved empty lacunar and BMD (100 mg/kg for 6 weeks, i.p. [31]) in STZ (65 mg/kg) induced diabetic rats. The results suggest that puerarin promotes osteoblast proliferation and inhibits osteoclast activation.

*Aralia echinocaulis* Hand-Mazz has been documented to distribute to kidney channel based on TCM theories [78]. The flavonoids isolated from the* Aralia echinocaulis* Hand-Mazz treatment (20 mg/kg) [32] significantly improved the bone metabolism evidenced by increasing BMD value, bone strength, and bone mineral content in STZ (30 mg/kg) insulted male rats. In addition, the aqueous extract of* Aralia echinocaulis* Hand-Mazz (3.6 g/kg for 14 days) was evidenced to promote the expressions of fibroblast growth factor receptor 2, Fms-like tyrosine kinase, and fetal liver kinase which contributed to promoting angiogenesis and osteoblastogenesis in fracture healing model rats [78].

Treatment with quercetin increased serum osteocalcin, ALP, and urinary deoxypyridinoline (quercetin at 5 mg/kg showed little effect, while quercetin at 30 mg/kg and 50 mg/kg exhibited good effects for 8 weeks) in STZ induced diabetic osteopenia in rats [33]. The beneficial effect of quercetin also reflected on partially reversing the decreased biomechanical quality and improving microarchitecture of the femurs in the diabetic rats. The underlying mechanism may be due to its antioxidant capability. In addition, quercetin was also demonstrated to have a protective role in reducing *β*-cell damage and decreasing glycaemia in diabetic rats [79]. Furthermore, quercetin is widely embraced in TCM herbs and possessed the function of improving blood circulation [80, 81].

The combination treatments with polyphenols extracted from the seeds of* Vitis vinifera* L. (0.028 mg/kg, every two days for 16 weeks) [34] and the fruits of* Sambucus nigra* L. (0.04 g/kg, every two days for 16 weeks) [35, 36] statistically improved BMD in STZ induced diabetic rats. Further, the combination treatments with* Nigella sativa* L. and parathormone [36, 37] have also been demonstrated to improve the bone strength in diabetic rats.

Taken together, the herbs used in the treatment of DOP animals usually have the functions of nourishing* Yin* and tonifying kidney, promoting blood circulation, which have been demonstrated to possess the ability of improving the bone metabolism in DOP [76, 82]. The most accepted pharmacologically active ingredients of these herbs are flavonoids, polyphenols, alkaloids, polysaccharides, and so forth.

### 4.2. The Advances of TCM Compounds in the Treatment of DOP in Animal Experiments

As is well known, TCM compounds are composed of 2 or more Chinese drugs, which may play a synergistic action during the treatment of diseases. An experiment performed by Du et al. [39] demonstrated that Bushen Zhuanggu capsule (16 and 32 g/kg) treatment not only decreased blood glucose but also increased bone calcium and phosphorus as well as bone minerals in alloxan (120 mg/kg) induced diabetic rats. Jiang et al. [40] also found that Shenxiaokang concentrated pill (modified from Bawei Shenqi pill) treatment increased the thickness of bone epiphysis and improved trabecular nesh structure in STZ (25 mg/kg) induced diabetic male rats. One formula named Bushen Jianpi Huoxue decoction [41] was also evidenced to significantly reduce blood glucose, phosphorus, and ALP level and improve insulin resistance as well as increase BMD value in high fat diet and STZ (30 mg/kg) induced rats. In addition, Shuanghuang Yigu formula treatment [42] significantly reduced blood glucose and urine desoxypyirdoxine levels and increased blood BGP level in ovariectomized rat with STZ (50 mg/kg) injection.

Emerging formulas have been demonstrated to play a beneficial role in management of DOP animals. Tangshukang [43], one formula invented by Guan et al., was demonstrated to improve calcium and phosphorus metabolism, increase BGP level, and reduce bone ALP level in STZ (60 mg/kg) induced diabetic osteoporosis rats. The underlying mechanism may be attributed to improve mRNA levels of vitamin D receptor and CBP9K in the small intestine and increase mRNA level of transforming growth factor- (TGF-) *β*1 in the bone [83].

Furthermore, Bushen Zhuanggu capsule [44] treatment improved blood glucose metabolism through regulating insulin secretion, promoting bone TGF-*β*1 expression and inhibiting caspase-3 expression in osteoblast, and modulating the metabolic disorder of calcium-regulating hormones such as PTH, calcitonin, and vitamin D3 in alloxan (i.p. of 120 mg/kg, twice every other day) induced diabetic rats.

In addition, Qianggubao treatment [45] reduced the expressions of interleukin-6 (IL-6), tumor necrosis factor alpha (TNF-*α*), and carboxyterminal cross-linked telopeptide of type I collagen (ICTP) and increased IGF-1 and carboxyterminal propeptide of type I procollagen (PICP) production in alloxan (i.p. of 120 mg/kg, twice every other day) induced diabetic osteoporosis rats. Further, Qianggubao [84] treatment also inhibited the formation of AGEs which was unfavorable for bone formation [85].

Most of formulas used in management of DOP not only have the function of improving bone metabolism but also afford effects on regulating glucose metabolism. However, as shown in Table 4, the ingredients of the formulas are not constant and fixable. And the commonly used TCM herbs also exhibit the effects of tonifying kidney, promoting blood circulation and removing blood stasis, which is also consistent with the main pathogenesis of DOP in TCM.

As is mentioned in Table 4, three types of DOP models are employed to evaluate the effect of TCM in the treatment of DOP, including STZ, alloxan, and STZ combined with OVX induced animal models. In addition, pigs are also used as the experimental DOP models [86].

## 5. The Toxicity of the Herbs

Toxicity is becoming a rising concern in the application of TCM in the clinical trials. Contrary to most of the modern toxicity data derived from animal experiments, the toxicities of TCM herbs in Chinese literature have been documented through clinical experiences. Before properly reviewing and studying the toxicities of the species by modern medical, pharmacological, and/or pharmaceutical sciences, the researchers must bear in mind that the isolated chemicals and the extracts from the herbs are not identical to the original herbs or formulas, while the traditional properties and indications of TCM herbs and formulas are the validated source of interpreting and extrapolating assessments of the toxicities. For knowing how to well understand the toxicities of TCM herbs, we suggest that the interested readers consult Dr. Leung's review [87].

In the above-mentioned species, several of them were evidenced to have toxicity issues for medicinal use.* Nigella sativa* L. extracts were demonstrated to possess the ability of lowering blood glucose in the healthy subjects. Therefore, caution must be excised when* Nigella sativa* L. extracts were applied to treat pregnant women, children, and diabetic patients [88]. For children,* Nigella sativa* L. at doses below 80 mg/kg was considered as safe dosages as there were no observed side effects being reported [89]. For detailed toxicity information of* Nigella sativa* L., we suggest that the readers consult Shuid et al.'s review [35].

It has been revealed that* Pueraria tuberosa* Linn. (Fabaceae) methanol extract of tubers showed LD50 at 227.5 mg in acute study in rats. For subchronic study, repeated challenges (5–100 mg/100 g, for 30 days) dose-dependently increased hepatic enzymes in blood, inflammatory cell infiltration, and hepatocellular necrosis. Kinetic study (single dose at 227.5 mg/100 g) revealed a decrease in GSH and an increase in free-radical generation [90, 91]. Puerarin, the major component of Radix* Puerariae*, has been demonstrated to cause hepatotoxicity as well as pathological changes in jejunum and spleen by high dose administration of puerarin freeze dried powder (100 and 200 mg/kg for 13 weeks,* i.v.*) to SD rats [92]. Interested readers are encouraged to consult Zhang et al.'s review [93].

The extracts of Radix* Astragali*, which consist of* Astragalus* polysaccharide and* Astragalus* membranaceus saponins, were evidenced safe without any distinct toxicity and side effects in the subchronic toxicity study. The safety dosage range is 2.85–19.95 g/kg for beagle dogs and 5.7–39.9 g/kg for rats [94]. And so far, no clinical toxicity data were reported.

The major tanshinones (cryptotanshinone, tanshinone I, tanshinone IIA, and dihydrotanshinone) isolated from* Salvia miltiorrhiza* Bunge were observed to induce HepG2 cell apoptosis in vitro [95]. Flow cytometry results demonstrated that tanshinone IIA (12.5 and 25 *μ*M) treatment increased fragmented DNA in HepG2 cells. Cryptotanshinone, tanshinone I, and dihydrotanshinone elevated GSH/GSSG ratio at low concentrations (1.56 and 3.13 *μ*M) and decreased GSH/GSSG ratio at high concentrations (6.25–25 *μ*M), which indicates that these compounds may disturb redox balance in HepG2 cells. Another experiment performed by Xu et al. claimed that liposoluble components of* Salvia miltiorrhiza* Bunge (IC50 = 2.181 *μ*g/mL) and tanshinone IIA (IC50 = 6.176 *μ*g/mL) inhibited proliferation of rat retinal Müller cell in vitro by CCK-8 examination at 48 h [96]. But the authors did not show the cell viabilities at various time points which are useful to evaluate the drug toxicity.

Cortex* Eucommiae* is also widely used in the treatment of DOP. In chronic toxicity experiments in rats, the seeds and barks of* Eucommia ulmoides* Oliv. were found to affect the utilization rate of food, the routine blood and liver function, and the organ coefficient of the liver, spleen, testis, and ovary. But the investigators did not find abnormalities in histological examination. They also did not discover abnormalities in acute toxicity, bone marrow of mice micronuclear test, and sperm deformity experiments [97, 98].

## 6. Conclusions and Remarks

**Scheme 1:**
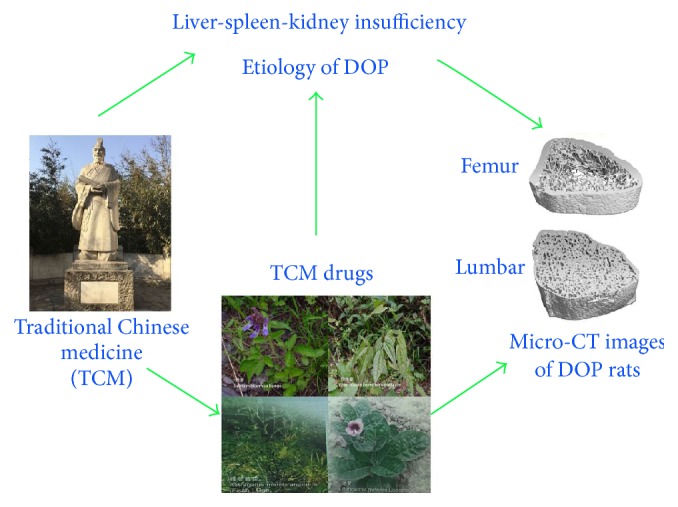
Liver-spleen-kidney insufficiency is closely associated with the induction of DOP. The most commonly used TCM herbs for treatment of DOP are Radix* Astragali*, Radix et Rhizoma* Salviae Miltiorrhizae*, Radix* Rehmanniae* Preparata, and Herba* Epimedii*. These herbs are evidenced to improve the management of DOP in clinical and preclinical study.

The clinical studies and basic research achievements support that TCM offers a new strategy in prevention and treatment of DOP (Scheme 1). TCM herbs and compounds have effects on resolving DOP through invigorating kidney* Qi*, promoting blood circulation, and removing blood stasis. This is consistent with TCM belief that liver-spleen-kidney insufficiency is closely associated with the induction of DOP. The most commonly used TCM herbs for treatment of DOP are Radix* Astragali*, Radix et Rhizoma* Salviae Miltiorrhizae*, Radix* Rehmanniae* Preparata, and Herba* Epimedii*. The investigators also demonstrate that these herbs have the beneficial effects on diabetes and osteoporosis. Three types of DOP models (STZ, alloxan, and STZ plus OVX induced animal disease models) are employed to evaluate the effect of TCM on DOP animals. These results clearly support that TCM herbs treatment not only improves bone metabolism but also prevents the development of diabetes. It is noticeable that some of TCM herbs and extracts may have the toxicities over the thresholds. Great care must be taken when TCM herbs are employed to treat DOP.

However, the basic research on single herbs and TCM compounds in the treatment of DOP is still very weak, and there is still a long way to find the mechanisms and active ingredients in TCM herbs. The researchers also need to pay attention that the pathogenesis of DOP is a multifaceted chronic metabolic disease, and a comprehensive study with multidisciplinary technology and comprehensive usage of various approaches should be employed to explore pathogenesis and to prevent the development of DOP. The further understanding of these concerns will contribute to providing a more scientific basis for management of DOP.

## Figures and Tables

**Table 1:** Clinical used TCM prescriptions for treatment of patients with DOP.

Compound prescription name	Treatment aim and biomarker examination	Results (# of patients)	References
Tanggukang decoction 7^a^	Fasting blood glucose (FBG), glycosylated hemoglobin (HbA1C), bone mineral density (BMD), superoxide dismutase (SOD), malondialdehyde (MDA), bone gla-protein (BGP), Ca and P in serum, and urine	FBG and HbA1C levels decreased; BMD, BGP, and serum SOD levels increased; serum MDA decreased; urine Ca and P decreased. Control treatment using oyster shell calcium chewable tablet	[5, 6]

Bushen Huoxue decoction 11^b^	Significant effect: BMD increased; clinical symptoms and pains disappeared. Effective: clinical symptoms alleviated; BMD remained the same. Ineffective: BMD and clinical symptoms remained the same	Markedly improved (24) Moderately improved (4) Ineffective (2) Overall efficacy: 93.3% Control treatment: Qianggu capsule	[7]

Bushen Tongluo decoction 9^c^	Broadband ultrasound attenuation (BUA), sound of speed (SOS), Ca and P in serum, alkaline phosphatase (ALP), FBG, and HbA1C	BUA and SOS significantly increased; FBG and HbA1C levels reduced. Control treatment using caltrate D	[8, 9]

Jiawei Qing'e Pill 9^d^	Ca, P/creatinine (Cr) ratios in blood and urine	Markedly improved (25) Moderately improved (11) Ineffective (4) Overall efficacy: 90% Serum Ca decreased by 0.48 mM/L; serum P increased by 0.44 mM/L; urine Ca/Cr ratio decreased by 0.29. Control treatment using alfacalcidol and caltrate D	[10]

Xihongkang 7^e^	Significant effect: clinical symptoms disappeared; FBG < 7.2 mM; two-hour postprandial blood glucose (2 hPG) < 8.3 mM; HbA1C < 6% or HbA1C decreased by *⩾*30%; BMD increased by *⩾*20%. Effective: clinical symptoms alleviated; FBG < 8.3 mM/L; 2 hPG < 10.0 mM/L; HbA1C < 8% or HbA1C decreased by *⩾*10%; BMD increased by *⩾*10%. Ineffective: no significant improvement in symptoms; HbA1C and BMD remained the same	Markedly improved (56) Moderately improved (68) Ineffective (16) Overall efficacy: 88.6% Control treatment using alfacalcidol, glipizide, and caltrate D	[11]

Yishen Zhuanggu compound 9^f^	Significant effect: pain disappeared; BMD increased by 0.05 g/cm^2^. Effective: pain significantly alleviated; BMD remained the same or decreased by <0.05 g/cm^2^. Ineffective: no improvement in symptoms	Markedly improved (14) Moderately improved (17) Ineffective (1) Overall efficacy: 96.8% Control treatment using alfacalcidol and caltrate D	[12]

Huqian pill 8^g^	Significant effect: symptom and physical sign disappeared; 23 mM/d ⩽ urine P ⩽ 48 mM/d; 2.5 mM/d ⩽ urine Ca ⩽ 7.5 mM/d; 16.7 mM/d ⩽ urine Mg^2+^ ⩽ 100 mM/d; 0.96 mM/L ⩽ serum P ⩽ 1.45 mM/L; 0.8 mM ⩽ serum Mg^2+^ ⩽ 1.2 mM. X-ray appeared normal. Effective: clinical symptoms alleviated; urine Ca remained 6.5 ± 2.5 mmol/d; urine P remained 38 ± 15 mmol/d; urine Mg^2+^ remained 80 ± 40 mmol/d; X-ray without obviously abnormal. Ineffective: no improvement in symptoms and signs as well as other biomarkers	Markedly improved (23) Moderately improved (28) Ineffective (5) Overall efficacy: 91.07% No controls	[13]

Gusong decoction 13^h^	Significant effect: symptoms and physical signs score decreased by *⩾*70%; BMD increased by *⩾*2%; significant improvement in bone metabolism index; FPG and 2 hPG returned to the normal or decreased by *⩾*40%; HbA1C ⩽ 6.2% or decreased by *⩾*30%. Effective: symptoms and physical signs score decreased between 30% and 70%; BMD increased between 1% and 2%; bone metabolism index improved; FPG and 2 hPG decreased by *⩾*20%; HbA1C decreased between 10% and 30%. Ineffective: no significant change in clinical symptoms; symptoms and physical signs score decreased by <30%; BMD increased by <1%; no improvement in bone metabolism index, FPG and 2 hPG, and HbA1C	Markedly improved (15) Moderately improved (22) Ineffective (3) Overall efficacy: 92.5% Control treatment using vitamin D_3_ calcium carbonate tablets and alendronate sodium tablets	[14]

Gusong decoction 4^i^	Significant effect: lumbar vertebra pain and other clinical symptoms disappeared or total scores decreased by more than 2/3. Effective: lumbar vertebra pain and other clinical symptoms markedly alleviated or total scores decreased by more than 1/3. Ineffective: no improvement in clinical symptoms; total scores did not reach the effective value	Markedly improved (21) Moderately improved (14) Ineffective (3) Overall efficacy: 92.11% Control treatment using glipizide and alendronate sodium tablets	[15]

Tangmaikang granules combined with alendronate sodium tablets 9^j^	Significant effect: lumbar vertebra pain disappeared; significant increase in BMD. Effective: lumbar vertebra pain alleviated with no significant increase in BMD. Ineffective: lumbar vertebra pain still existed; BMD remained the same	Markedly improved (24) Moderately improved (21) Ineffective (3) Overall efficacy: 93.75% Control treatment using alendronate sodium tablets	[16]

Bushen Huoxue decoction 0^k^	Significant effect: back pain disappeared; symptoms and physical signs score decreased by 2/3; significant improvement in bone metabolism index; BMD increased by more than 0.06 g/cm^2^. Effective: back pain obviously alleviated; symptoms and physical signs scores decreased by 1/3; no change or aggravation was found in the biomarkers and BMD. Ineffective: no improvement in all the aspects	Markedly improved (3) Moderately improved (26) Ineffective (1) Overall efficacy: 96.7% Control treatment using caltrate D	[17]

Jiawei Gutongxian capsule 14^l^	Cure: lumbar vertebra pain disappeared; X-ray appeared normal. Significant effect: lumbar vertebra pain obviously alleviated; significant improvement in X-ray. Effective: lumbar vertebra pain alleviated; improvement in X-ray. Ineffective: lumbar vertebra pain and X-ray remained the same	Cure (1) Markedly improved (8) Moderately improved (27) Ineffective (4) Overall efficacy: 90.0% Control treatment using osteoform capsule	[18]

Qianggubao capsule 8^m^	Back pain; BMD	Markedly improved (24) Moderately improved (13) Ineffective (3) Overall efficacy: 92.5%	[19]

Jiangu formula 12^n^	Clinical symptomatic scales	Markedly improved (18) Moderately improved (28) Ineffective (4) Overall efficacy: 92.0% Control treatment using osteoform capsule	[20]

Jiangtang Bushen formula 10^o^	BMD, serum C-reactive protein (CRP)	BMD and serum CRP significantly increased. Control treatment using caltrate D	[21]

Huolisu oral liquid 5^p^	Significant effect: clinical symptoms markedly improved; BMD significantly increased. Effective: clinical symptoms alleviated; BMD slightly increased. Ineffective: clinical symptoms and BMD remained the same	Markedly improved (12) Moderately improved (86) Ineffective (14) Overall efficacy: 79.1% Control treatment using salmon calcitonin	[22]

Migu decoction 7^q^	Visual analogue scale (VAS) pain score, BMD	VAS pain score significantly improved; BMD significantly increased. Control treatment using calcium carbonate	[23]

Xianling Gubao 6^r^	Clinical symptom scales and BMD	Clinical symptom scales and BMD significantly improved. Control treatment using caltrate D	[24]

^a^Radix *Rehmanniae* Preparata, Fructus *Corni*, Rhizoma *Dioscoreae*, Herba *Cynomorii*, *Carapax et Plastrum Testudinis*, Rhizoma *Chuanxiong*, and Radix et Rhizoma *Salviae Miltiorrhizae* (dosage information is not available).

^b^Radix *Astragali* 30 g, Herba *Epimedii* 12 g, Rhizoma *Polygonati* 12 g, Colla *Cornus Cervi* (melting in boiled water) 15 g, Rhizoma *Dioscoreae* 30 g, Semen* Astragali Complanati* 15 g, Radix *Puerariae *Lobatae 30 g, Radix *Polygoni* Multiflori 15 g, Radix et Rhizoma *Salviae Miltiorrhizae* 30 g, Radix et Rhizoma *Rhei* 10 g, and *Sanguis Draconis* 10 g.

^c^Radix *Astragali*, Colla *Cervi Cornus*, Radix *Rehmanniae*, Fructus *Psoraleae*, Herba *Epimedii*, Fructus *Ligustri Lucidi*, Radix *Notoginseng*, Radix et Rhizoma *Salviae Miltiorrhizae*, and *Pheretima* (dosage information is not available).

^d^Cortex *Eucommiae* 12 g, Fructus *Psoraleae* 12 g, Radix *Astragali* 30 g, Radix *Notoginseng* 15 g, Rhizoma *Dioscoreae* 15 g, Colla *Cornus Cervi* (melting in boiled water) 6 g, Radix et Rhizoma *Salviae Miltiorrhizae* 12 g, *Os Draconis* 30 g, and *Concha Ostreae* 30 g.

^e^Radix *Astragali*, Radix et Rhizoma *Rhei*, Fructus *Ligustri Lucidi*, Fructus *Lycii*, Hirudo, Flos *Carthami*, and Radix et Rhizoma *Rhodiolae Crenulatae* (dosage information is not available).

^f^Radix *Rehmanniae* Preparata 15 g, Fructus *Corni* 10 g, Rhizoma *Chuanxiong* 15 g, Rhizoma *Drynariae* 15 g, Fructus *Psoraleae* 15 g, Herba *Epimedii* 12 g, Radix et Rhizoma *Salviae Miltiorrhizae* 15 g, Radix* Cyathulae* 15 g, and Fructus *Lycii* 20 g.

^g^Cortex *Phellodendri Chinensis* 10 g, Radix *Rehmanniae* Preparata 15 g, *Carapax et Plastrum* Testudinis 15 g, Radix *Paeoniae* Alba 12 g, Rhizoma *Anemarrhenae* 9 g, *Pericarpium Citri Reticulatae* 10 g, Herba *Cynomorii *10 g, and Rhizoma *Zingiberis *4 g.

^h^Radix *Astragali*, Radix *Codonopsis*, Radix *Polygoni Multiflori*, Radix *Angelicae Sinensis*, Rhizoma *Atractylodis Macrocephalae*, *Poria*, *Carapax et Plastrum* Testudinis, *Carapax Trionycis*, Herba *Taxilli*, Herba *Epimedii*, Radix *Dipsaci*, Cortex *Eucommiae*, and Fructus *Aurantii* (dosage information is not available).

^i^Rhizoma *Drynariae* 20 g, Herba *Epimedii* 20 g, Rhizoma *Atractylodis Macrocephalae* 15 g, and Cortex *Eucommiae* 15 g.

^j^Radix *Astragali*, Radix *Rehmanniae*, Radix *Paeoniae Rubra*, Radix et Rhizoma *Salviae Miltiorrhizae*, Radix *Cyathulae*, Radix *Ophiopogonis*, Rhizoma *Polygonati*, Herba *Epimedii*, and Radix *Puerariae* Lobatae (dosage information is not available).

^k^Prescription constituents and dosage information are not available.

^l^Radix *Rehmanniae* Preparata, Radix *Morindae* Officinalis, Herba *Cistanches*, Rhizoma *Drynariae*, *Pyritum*, Rhizoma *Curculiginis*, Herba *Epimedii*, Radix *Angelicae* Sinensis, Rhizoma *Anemarrhenae*, Radix *Notoginseng*, *Carapax et Plastrum Testudinis*, *Placenta Hominis*, Radix *Astragali*, and *Endothelium Corneum Gigeriae* Galli (dosage information is not available).

^m^Radix *Astragali*, Fructus *Corni*, Herba *Dendrobii*, Rhizoma *Dioscoreae*, Rhizoma *Drynariae*, Colla Cornus *Cervi* (melting in boiled water), Radix et Rhizoma* Salviae Miltiorrhizae*, and *Concha Ostreae* (dosage information is not available).

^n^Herba *Epimedii *10 g, Herba *Cistanches* 10 g, Cortex *Eucommiae* 18 g, Radix *Dipsaci* 12 g, Radix *Polygoni Multiflori* 10 g, Radix Rehmanniae *Preparata* 12 g, Rhizoma *Dioscoreae* 15 g, Fructus *Corni* 10 g, Fructus *Lycii* 12 g, Radix *Angelicae Sinensis* 10 g, Radix *Cyathulae* 18 g, and *Poria* 12 g.

^o^Radix* Astragali* 30 g, Radix *Paeoniae Rubra* 20 g, Radix et Rhizoma *Salviae Miltiorrhizae* 20 g, Cortex *Mori* 10 g, Radix *Trichosanthis* 20 g, Fructus *Lycii* 15 g, Radix *Scrophulariae* 20 g, Radix *Rehmanniae* 20 g, Semen *Cuscutae* 15 g, and Rhizoma *Drynariae* 15 g (modification according to symptoms. If diagnosed as Qi deficiency, Radix* Pseudostellariae* (30 g) is added to the above prescription and the dosage of Radix *Astragali* is modified from 30 g to 50 g. If diagnosed as Yin deficiency, Herba *Dendrobii *(15 g) is added to the above prescription. If diagnosed as blood deficiency, Radix* Paeoniae Alba* (20 g) is added to the above prescription. If diagnosed as blood stasis, Rhizoma *Chuanxiong* (20 g) is added to the above prescription. If diagnosed as phlegm-heat, Fructus *Trichosanthis* (15 g) and Caulis *Bambusae* in *Taenia* (10 g) are added to the above prescription).

^p^Herba *Epimedii*, Radix et Rhizoma S*alviae Miltiorrhizae*, Radix *Astragali*, Radix *Ophiopogonis*, and Fructus* Lycii *(dosage information is not available).

^q^Radix *Rehmanniae Preparata* 15 g, Herba *Epimedii* 10 g, Fructus *Psoraleae* 15 g, Radix *Notoginseng* 15 g, Rhizoma *Drynariae* 15 g, Semen *Cuscutae* 15 g, and Rhizoma *Dioscoreae *12 g.

^r^Herba *Epimedii*, Radix *Dipsaci*, Fructus *Psoraleae*, Radix *Rehmanniae*, Radix et Rhizoma *Salviae Miltiorrhizae*, and Rhizoma *Anemarrhenae* (dosage information is not available).

**Table 2:** Medicinal herbs most frequently used in clinical trials of DOP.

Name of herb	Frequency
Radix *Astragali*	11
Herba *Epimedii*	10
Radix et Rhizoma *Salviae Miltiorrhizae*	10
Radix *Rehmanniae* Preparata	6
Rhizoma *Drynariae*	6
Fructus *Lycii*	5
Cortex *Eucommiae*	4
Rhizoma *Dioscoreae*	4

**Table 3:** Single herb or herbal extracts used in the treatment of DOP animals.

TCM name	Active constituents	Animal model	Administration route, duration, and dosage	Reference
Fructus *Ligustri Lucidi*	Water fraction of Fructus *Ligustri Lucidi* ethanol extract	STZ (40 mg/Kg)	Intragastric administration (i.g.) (574 mg/kg) for 6 weeks	[25]
Radix et Rhizoma *Salvia miltiorrhiza*	—	Alloxan (200 mg/kg)	i.g. (5 g/kg) for 8 weeks	[26, 27]
Rhizoma *Chuanxiong*	Tetramethylpyrazine	STZ (60 mg/Kg)	i.g. (100 mg/kg) for 15 weeks	[28]
Cortex *Eucommiae*	Ethanol extracts of *Eucommia ulmoides* Oliv. leaves	STZ combined with OVX	i.g. (6 g/kg) for 8 weeks	[29]
Radix *Puerariae*	Puerarin	STZ (65 mg/Kg)	i.g. (100 mg/kg) for 6 weeks	[30, 31]
*Aralia echinocaulis* Hand-Mazz	The flavonesof *Aralia echinocaulis *Hand-Mazz	STZ (30 mg/kg)	i.g. (20 mg/kg) for 6 weeks	[32]
—	Quercetin	STZ	i.g. (5, 30 and, 50 mg/kg) for 8 weeks	[33]
*Vitis vinifera *L. seeds	—	STZ (60 mg/kg)	i.g (0.028 mg/kg every other day) for 16 weeks	[34]
*Nigella sativa* L.	Thymoquinone	STZ (50 mg/kg)	i.g. (2 mL/kg) for 4 weeks	[35–38]

Note: “—” denotes that the content was not clearly stated in the cited reference.

**Table 4:** TCM prescriptions in the treatment of DOP animals.

TCM compound prescription name	Animal model	Administration route, duration, and dosage	Reference
Bushen Zhuanggu capsule 2^a^	Alloxan (120 mg/kg, twice every other day)	i.g. (8, 16 and 32 g/kg) for 8 weeks	[39]
Shenxiaokang concentrated pill 9^b^	STZ (25 mg/kg), once a day for 5 days	i.g. (1.58 g/200 g) for 7 weeks	[40]
Bushen Jianpi Huoxue decoction 8^c^	high fat diet for 4 weeks, i.p. of STZ (30 mg/kg)	i.g. (15 g/kg) for 12 weeks	[41]
Shuanghuang Yigu formula 9^d^	i.p. of STZ (50 mg/kg) combined with OVX	i.g. (11.5 g/kg) for 10 weeks	[42]
Tangshukang capsule 0^e^	i.p. of STZ (60 mg/kg)	i.g. (15 g/kg) for 12 weeks	[43]
Bushen Zhuanggu capsule 0^f^	i.p. of alloxan (120 mg/kg, twice every other day)	i.g (8, 16 and 32 g/kg) for 7 weeks	[44]
Qianggubao 10^g^	i.p. of alloxan (120 mg/kg, twice every other day)	i.g (1 mL/100 g) for 12 weeks	[45]

^a^Radix *Rehmanniae* Preparata and *Carapax et Plastrum Testudinis* (dosage information is not available).

^b^Radix *Rehmanniae* Preparata, Rhizoma *Dioscoreae*, Fructus *Corni*, *Poria*, Rhizoma *Alismatis*, *Pericarpium Citri* Reticulatae, Radix *Astragali*, and *Hirudo* (dosage information is not available).

^c^Radix *Rehmanniae* Preparata, Cortex *Eucommiae*, Radix *Astragali*, Fructus *Lycii*, *Colla Cornus* Cervi (melting in boiled water), Radix et Rhizoma *Salviae Miltiorrhizae*, Rhizoma *Anemarrhenae*, and Radix *Cyathulae* (dosage information is not available).

^d^ *Os Draconis*, Radix *Rehmanniae* Preparata, Radix *Astragali*, Cortex *Eucommiae*, Radix *Dipsaci*, Rhizoma *Drynariae*, Fructus *Lycii*, Fructus *Corni*, *Poria*, Radix *Angelicae* Sinensis, Radix *Cyathulae*, Herba *Artemisiae Anomalae* (dosage information is not available).

^e^TCM names and dosage information are not available.

^f^TCM names and dosage information are not available.

^g^Radix *Astragali*, Herba *Dendrobii*, Radix et Rhizoma *Salviae Miltiorrhizae*, Cortex *Eucommiae*, Fructus *Psoraleae*, Fructus *Corni*, Rhizoma *Dioscoreae*, Rhizoma *Drynariae*, *Colla CornusCervi* (melting in boiled water), and *Concha Ostreae* (dosage information is not available).
